# Specific sub-regions along the longitudinal axis of the hippocampus mediate antidepressant-like behavioral effects

**DOI:** 10.1016/j.ynstr.2021.100331

**Published:** 2021-04-22

**Authors:** Brunno Rocha Levone, Gerard M. Moloney, John F. Cryan, Olivia F. O'Leary

**Affiliations:** a Department of Anatomy and Neuroscience, University College Cork, Cork, Ireland; b APC Microbiome Ireland, University College Cork, Cork, Ireland

**Keywords:** Intermediate hippocampus, Ventral hippocampus, Antidepressant, Depression, Anxiety

## Abstract

Current antidepressants are suboptimal due incomplete understanding of the neurobiology underlying their behavioral effects. However, imaging studies suggest the hippocampus is a key brain region underpinning antidepressant action. There is increasing attention on the functional segregation of the hippocampus into a dorsal region (dHi) predominantly involved in spatial learning and memory, and a ventral region (vHi) which regulates anxiety, a symptom often co-morbid with depression. However, little is known about the roles of these hippocampal sub-regions in the antidepressant response. Moreover, the area between them, the intermediate hippocampus (iHi), has received little attention. Here, we investigated the impact of dHi, iHi or vHi lesions on anxiety- and depressive-like behaviors under baseline or antidepressant treatment conditions in male C57BL/6 mice (n = 8–10). We found that in the absence of fluoxetine, vHi lesions reduced anxiety-like behavior, while none of the lesions affected other antidepressant-sensitive behaviors. vHi lesions prevented the acute antidepressant-like behavioral effects of fluoxetine in the tail suspension test and its anxiolytic effects in the novelty-induced hypophagia test. Intriguingly, only iHi lesions prevented the antidepressant effects of chronic fluoxetine treatment in the forced swim test. dHi lesions did not impact any behaviors either in the absence or presence of fluoxetine. In summary, we found that vHi plays a key role in anxiety-like behavior and its modulation by fluoxetine, while both iHi and vHi play distinct roles in fluoxetine-induced antidepressant-like behaviors.

## 1 Introduction

Depression is the current leading cause of disability worldwide (WHO, 2017). Half of depressed patients do not respond to first-line antidepressant treatment, and a third remain treatment-resistant (Trivedi et al., 2006). Antidepressant drug development has been hampered by our incomplete understanding of the neurobiology underlying the pathophysiology of depression and its successful treatment. However, human neuroimaging studies implicate the hippocampus as a key area of the brain involved in depression (Bremner et al., 2000, Frodl et al., 2002, Sheline et al., 1996, Sheline et al., 2003). In parallel, studies in animals report that chronic stress, a depression risk factor, induces hippocampal atrophy (Lee et al., 2009; Tse et al., 2014) and decreases adult hippocampal neurogenesis (Gould et al., 1997; Tanti and Belzung, 2013b; Levone et al., 2015; Dioli et al., 2017; Alves et al., 2018), an effect reversed by chronic antidepressant treatment including the selective serotonin reuptake inhibitor (SSRI), fluoxetine (Malberg et al., 2000; Malberg and Duman, 2003; Tanti et al., 2012).

Accumulating evidence suggests that the hippocampus is functionally segregated along its longitudinal axis into anterior and posterior regions in humans, and corresponding ventral (vHi) and dorsal (dHi) regions in rodents. Indeed, lesions of the dHi but not vHi have been shown to impair spatial learning and memory in rodents (Moser et al., 1995; Bannerman et al., 2002; Pothuizen et al., 2004). On the other hand, it has been reported that the vHi plays a role in the stress response (Fanselow and Dong, 2010; Tanti and Belzung, 2013a; O’Leary and Cryan, 2014; Levone et al., 2015; Anacker et al., 2018; Floriou-Servou et al., 2018; Levone et al., 2020), anxiety (Bannerman et al., 2002, Bannerman et al., 2003, Bannerman et al., 2004, Felix-Ortiz and Tye, 2014) and social behaviors (Bannerman et al., 2002; Felix-Ortiz and Tye, 2014; Okuyama et al., 2016). Studies have also reported that chronic antidepressant treatment increases adult hippocampal neurogenesis predominantly in the vHi rather than the dHi (Jayatissa et al., 2006; Tanti et al., 2012; O’Leary and Cryan, 2014) and increases neural progenitor cell proliferation in the human anterior hippocampus (Boldrini et al., 2009, Boldrini et al., 2012), thus suggesting that the vHi is an important site of antidepressant action. However, the relative roles of the vHi versus the dHi in antidepressant-induced behavior remains to be elucidated.

**Fig. 1:**
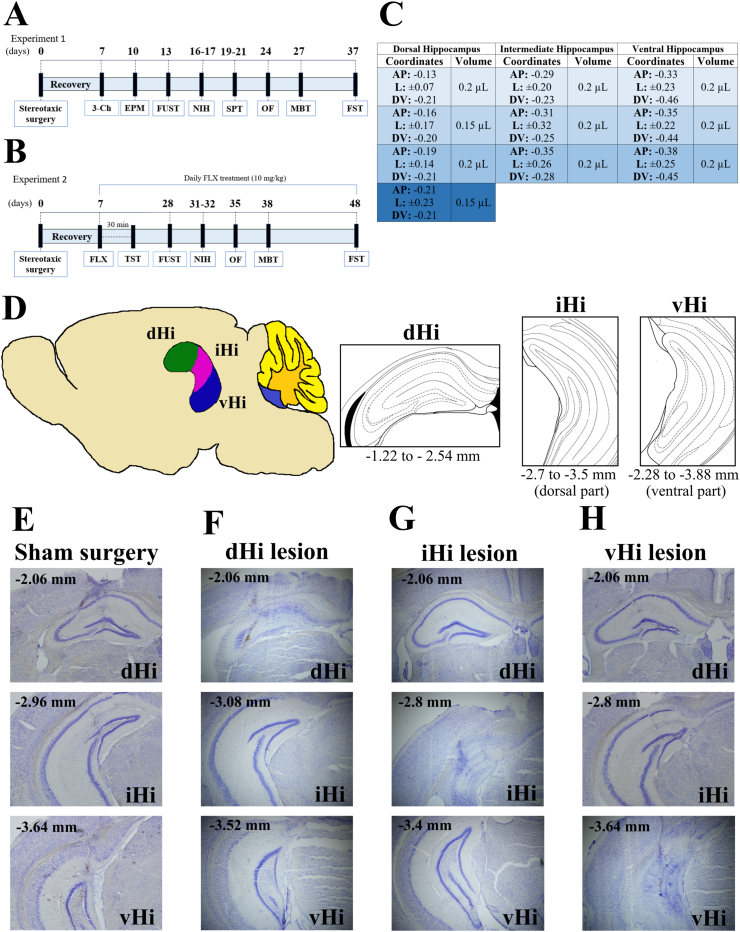
Dorsal (dHi), intermediate (iHi) or ventral (vHi) hippocampus were lesioned and animals underwent a battery of behavioral testing with or without chronic treatment with fluoxetine. (A) Schematic of the experiment examining the impact of the dHi, iHi or vHi lesions on behavior. (B) Schematic of experiment examining the impact of dHi, iHi or vHi lesions on fluoxetine-induced changes in behavior (C) Stereotaxic coordinates and injection volumes used to bilaterally lesion the dHi, iHi or vHi with ibotenic acid. (D) Illustration of co-ordinates used to define the dHi, iHi and vHi (E–H) Representative images of cresyl violet stained dHi, iHi and vHi from sham surgery animals and dHi-, iHi- and vHi-lesioned animals. Coordinates in the representative images are in relation to bregma (AP). (E) dHi, iHi and vHi sham groups. (F) dHi lesion, showing that iHi and vHi remain intact. (G) iHi lesion, showing that dHi and iHi remain intact. (H) vHi lesion, showing that dHi and iHi remain intact. Abbreviations: 3-Ch: three-chambers test, AP: anteroposterior, DV: dorsoventral, EPM: elevated plus maze, FLX: fluoxetine, FST: forced swim test, FUST: female urine sniffing test, L: lateral, MBT: marble burying test, NIH: novelty-induced hypophagia, OF: open field, SPT: saccharin preference test, TST: tail suspension test. (For interpretation of the references to colour in this figure legend, the reader is referred to the Web version of this article.)

The hippocampus exhibits a gradient of gene expression and diverse neurotransmitter receptors densities along its longitudinal axis (Lothmann et al., 2020), thus the concept of an intermediate sub-region (iHi) has been introduced (Bast et al., 2009, Cembrowski et al., 2016a, Cembrowski et al., 2016b, Leonardo et al., 2006, Levone et al., 2020, Strange et al., 2014, Thompson et al., 2008). We recently demonstrated that postnatal neural progenitor cell-derived neurons from the vHi are more sensitive to longer-term exposure to glucocorticoids than those derived from the dHi, while those from the iHi show an intermediate effect, suggesting a gradient-like effect (Levone et al., 2020). While the iHi has overlapping characteristics with both the dHi and vHi (Cenquizca and Swanson, 2007), it might also be functionally independent (Bast et al., 2009), but its roles in anxiety-like and depression-like behavior under homeostatic conditions and more specifically in the antidepressant response have yet to be determined. Here, using selective excitotoxic-induced lesions of the mouse iHi as well as the dHi or vHi, we investigate the specific roles of the iHi, dHi, and vHi in the regulation of anxiety-like and depressive-like behaviors under baseline conditions (Fig. 1A). Moreover, we also determine whether the beneficial effects of antidepressants are mediated by these specific hippocampal sub-regions by investigating the impact of selective excitotoxic-induced lesions of the mouse iHi, dHi or vHi on the regulation of anxiety-, depressive-, and antidepressant-like behaviors by chronic treatment with the antidepressant, fluoxetine (Fig. 1B).

## 2 Methods and materials

### 2.1 Experimental design

We first examined the roles of the dHi, iHi and vHi in social behavior, anxiety, anhedonia and antidepressant-like behavior (*Experiment 1*; Fig. 1A). Mice underwent either sham surgery (saline microinjections) or ibotenic-induced lesions of dHi, iHi, or vHi (3–4 bilateral ibotenic acid injections, as shown in Fig. 1C). Seven days following surgery, animals underwent a battery of behavioral tests as shown in Fig. 1A. The experimental groups, final sample number (after excluding misplaced lesions), surgery success rate and number of misplaced lesions/excluded animals were as follows: (1) dHi-sham, n = 10 (83.3% success, 2 excluded); (2) dHi-lesion, n = 8 (66.7% success, 4 excluded); (3) iHi-sham, n = 9 (75% success, 3 excluded); (4) iHi-lesion, n = 9 (57.1% success, 6 excluded); (5) vHi-sham, n = 10 (55.6% success, 8 excluded) and (6) vHi-lesion, n = 9 (52.9% success, 8 excluded).

Given that diverse hippocampal sub-regions showed differential roles on the regulation of emotional behaviors, especially in the novelty-induced hypophagia test, a hippocampal neurogenesis- and antidepressant-responsive test, we then examined the roles of the dHi, iHi and vHi in mediating the antidepressant and anxiolytic behavioral effects of fluoxetine treatment (*Experiment 2*; Fig. 1B). Ibotenic-induced lesions of the dHi, iHi and vHi of the mouse hippocampus were performed, while some animals underwent a sham surgery. Some animals underwent a surgery (Sham Surgery) without the injection of saline (as they serve as a control to the three sub-regions). Seven days following surgery, animals were injected with either saline (sham-saline) or fluoxetine (all other groups; 10 mg/kg i.p. per day, PHR1394, Sigma-Aldrich) daily for 21 days and then underwent a battery of behavioral tests while also receiving daily fluoxetine (or saline) injections as shown in Fig. 1B. 30 min after the first injection, animals underwent the tail suspension test, to assess the effects of acute fluoxetine treatment. The impact of lesions and chronic fluoxetine treatment on acute swim stress-induced increases in plasma corticosterone concentrations was also investigated (data not shown). The experimental groups, final sample numbers (after excluding misplaced lesions), surgery success rate and number of misplaced lesions/excluded animals were: (1) sham-saline, n = 10 (100% success, 0 excluded); (2) dHi sham-fluoxetine, n = 10 (83.3% success, 2 excluded); (3) dHi lesion-fluoxetine, n = 10 (83.3% success, 2 excluded); (4) iHi sham-fluoxetine, n = 9 (81.8% success, 2 excluded); (5) iHi lesion-fluoxetine, n = 10 (83.3% success, 2 excluded); (6) vHi sham-fluoxetine, n = 8 (66.7% success, 4 excluded) and (7) vHi lesion-fluoxetine, n = 8 (72.7% success, 3 excluded).

### 2.2 Animals

Male C57BL/6 mice (Envigo, UK) aged 8-weeks old, were housed in groups of 3–4 at arrival in a temperature controlled (21 ± 2 °C) room and allowed to acclimatize to the holding room for 7-days prior to initiation of experiments. Laboratory chow and water were provided ad libitum on a 12/12 h light/dark cycle (lights on at 7:30 a.m.). All procedures were conducted with approval from the Animal Experimentation Ethics Committee (AEEC) at University College Cork, under Individual Authorizations and a Project Authorization AE19130/P027, approved the Health Products Regulatory Authority (HPRA) Ireland and in accordance with the recommendations of the European Parliament and the Council of the EU Directive (2010/63/EU).

### 2.3 Stereotaxic surgery

Mice (20–26g) were anesthetized with isoflurane (5% induction, 1.7–2.5% maintenance) and received analgesia (5 mg/kg, s.c., Carprofen, Norbrook) prior to being placed in a stereotaxic frame. Fig. 1C shows the definition of dHi, iHi and vHi in coordinates and the sites for injection. As shown in Fig. 1D, small volumes (0.15–0.2 μl) of ibotenic acid (10 mg/ml, I2765, Sigma-Aldrich) diluted in PBS (pH 7.4) were injected bilaterally into either the dHi, iHi or vHi using coordinates from the Paxinos and Franklin's atlas for mouse brain (Paxinos and Franklin, 2012). Ibotenic acid causes excitotoxicity, damaging cells without affecting passaging fibers. Sham surgeries involved opening holes at the relevant areas of the skull but not injecting anything. Holes in the skull were covered with Bone-Wax (SMI, Z046) and the skin was sutured (Mersilk Suture 4–0, W329). An analgesic (Carpofen, Norbrook, 20 μg/ml) was added to the drinking water for 2 days post-surgery.

At the end of the behavioral experiments, mice were deeply anesthetized with pentobarbital (250 mg/kg, i.p., Euthanal, Merial) and transcardially perfused with ice-cold PBS followed by 4% PFA. Brains were removed, post-fixed in 4% PFA overnight, cryoprotected in 30% sucrose and then frozen at −80 °C. Brains were sectioned coronally (at 35 μm) through the entire rostro-caudal axis of the hippocampus and collected onto gelatinized slides. Sections were stained with cresyl violet (0.1%, Sigma-Aldrich, C5042). Injection sites were identified by light microscopy examination by an experimenter blind to the experimental treatment groups. Only animals with confirmed lesions in at least 75% of the brain sections from the specific sub-region were included (see representative images of shams in Fig. 1E and of lesions in Fig. 1F–H). Animals with only unilateral lesions or with lesions in multiple hippocampal sub-regions (e.g. both iHi and vHi) were excluded. Most excluded animals were from the vHi group due to the greater difficulty in precisely targeting this area compared with the dHi and iHi.

### 2.4 Fluoxetine treatment

One week after stereotaxic surgery, animals from the fluoxetine experiment (Fig. 1B) received daily injections with fluoxetine 10 mg/kg i.p. (except for Sham surgery group, which received saline injections).

### 2.5 Behavioral testing

One week after surgery, or immediately after fluoxetine treatment, animals underwent a battery of behavioral tests, including tests for sociability (three-chambers test), anxiety (elevated plus maze, marble burying test, novelty-induced hypophagia and open field), and depressive-like behaviors (female urine sniffing test, saccharin preference test, forced swim test and tail suspension test) (Fig. 1A and B).

#### 2.5.1 Three-chambers test of sociability and social novelty preference

Sociability and preference for social novelty were assessed in a three-chamber apparatus as previously described (Desbonnet et al., 2014). The apparatus consists of a box with three chambers (left and right and a smaller center chamber) with small circular openings allowing easy free access to all compartments. The test is composed of three sequential 10 min trials or stages. In the first stage, the test animal is allowed to explore the three empty chambers (habituation). The second stage tests sociability whereby an unfamiliar animal is placed in an inner wire mesh cage in one chamber and an object is placed in a wire mesh cage in the other chamber. The final stage, tests social novelty preference whereby the object is replaced by a novel animal. All animals were age- and sex-matched and each chamber was cleaned and lined with fresh bedding between trials. In each of the three stages, behavior was recorded by a video camera mounted above the apparatus and the time spent in each chamber was measured in each individual trial. In the second stage, data are expressed both as time spent with animal versus object and % preference for the animal (as a measure of sociability). In the third stage, data are expressed both as time spent with novel versus familiar animal and % preference for social novelty.

#### 2.5.2 Tests of anxiety

##### 2.5.2.1 Elevated plus maze (EPM)

The elevated plus maze is a test used to measure anxiety-like behavior (File et al., 2004; Sweeney et al., 2014). Each animal was placed in the center of the elevated plus maze (a cross shaped maze with 2 open arms and 2 closed arms, elevated from the ground) and their behavior was monitored and tracked for 5 min. Time and percentage of time and number of entries in the open and closed arms were analyzed. Mice tend to spend more time in the protected closed arms than the open arms. Thus, an increase in the percentage of time spent in the open versus closed arms is interpreted as reduced anxiety behavior.

##### 2.5.2.2 Novelty-induced hypophagia (NIH)

The novelty-induced hypophagia test is a test of anxiety sensitive to chronic antidepressant treatment (Dulawa and Hen, 2005; O’Leary et al., 2013). In this test, mice were trained to drink a diluted solution of sweetened condensed milk (3:1, water to milk) from a 10 ml serological pipette through the lid of the cage for 30 min per day for 3 days. On the fourth day, the latency to drink the milk in their home cage was measured. On the fifth day, mice were placed in a novel brightly lit cage (1200 lux) without bedding, and their latency to drink the milk was recorded. Placement in the novel cage increases the latency to drink the milk which is taken as an index of anxiety. Chronic antidepressant treatment decreases this latency. The data are presented as latency difference (i.e. the latency to drink in the novel cage minus latency to drink in the home cage).

##### 2.5.2.3 Marble burying test (MBT)

The marble burying test is used to measure phenotypes related to anxiety and obsessive compulsive-like behavior (Deacon, 2006). A cage (35 × 28 cm) was filled with clean corncob bedding overlaid with 20 glass marbles equidistant from each other in a 4 × 5 arrangement (Savignac et al., 2014). Mice were placed in the cage for 30 min and the number of marbles that are more than 2/3 buried were counted. An increase in the number of marbles buried is considered to be a proactive response to an anxiogenic stimulus and has also been described as a compulsive behavior to relieve anxiety (Deacon, 2006). Chronic fluoxetine treatment has been shown to decrease the number of marbles buried (Greene-Schloesser et al., 2011).

##### 2.5.2.4 Open field test (OF)

The open field test was used to measure locomotor activity as well as anxiety-like behavior. This test consisted of a rectangle box (Perspex sides and base: 40 × 33 cm^2^) in a dimly lit room (4 lux). Mice were placed in the center of the open field and allowed to explore the apparatus for 10 min. Locomotor activity was recorded by a camera placed over the open field and the distance travelled was automatically analyzed using Ethovision 11.5 software. The time spent in the center quarter (10 × 8.25 cm^2^) of the chamber was assessed as an index of anxiety behavior.

#### 2.5.3 Tests of anhedonia

##### 2.5.3.1 Female urine sniffing test (FUST)

The female urine sniffing test assesses anhedonia (a reduction in the ability to experience pleasure) which is a core feature of depression (Malkesman et al., 2010; Finger et al., 2011; O’Leary et al., 2014). This test takes advantage of the fact that rodent males are attracted to pheromonal odors from the opposite sex and thus is used in male mice as an index of sexual interest. Before the test, mice were transferred to a dimly lit (4 lux) room and habituated to the presence of a cotton tip applicator in their home cage for 1 h. Following habituation, mice were immediately presented with a cotton tip applicator with 60 μL of sterile water for a period of 3 min and the time spent sniffing the water was measured. 45 min later, a cotton tip with 60 μL of fresh urine from female mice in estrus was presented to the mice for 3 min and the time spent sniffing the urine was measured. Data are presented as the percentage of time spent sniffing urine over the total time spent sniffing urine plus water.

##### 2.5.3.2 Saccharin preference test (SP)

The saccharin preference test (Harkin et al., 2002) was used as another test for anhedonia. Mice were given access to both a water bottle and a saccharin solution (0.1%) bottle for a total period of 48 h. Cumulative saccharin and water intake were measured every 12 h (at 7:30 am and 7:30 p.m. each day). Every 12 h, the position of the bottles was reversed to avoid the development of preference for drinking from a particular side of the cage. Mice normally show a preference to consume the saccharin over water and chronic antidepressant treatment counteracts stress-induced decreases in this preference for saccharin (Harkin et al., 2002). The data are expressed as the cumulative percentage preference for saccharin over water [(saccharin consumption volume/water consumption volume) * 100].

#### 2.5.4 Tests of antidepressant-like behavior

##### 2.5.4.1 Tail suspension test (TST)

The tail suspension test is a test of antidepressant-like activity and behavioral despair (Steru et al., 1985; O’Leary and Cryan, 2009). In this test, the mouse was suspended by the tail from a horizontal bar for 6 min. Initially, the mouse displays escape-oriented behaviors but after several minutes adopts an immobile posture. Antidepressant drugs are known to decrease the time the mouse spends immobile in this test. In the present experiment, a single dose of fluoxetine was given 30 min prior to the start of the test to examine the role of hippocampal sub-regions in the acute antidepressant-like effects of fluoxetine. The data are expressed as time spent immobile.

##### 2.5.4.2 Forced swim test (FST)

The forced swim test is the most widely used experimental paradigm to assess antidepressant drug-like activity (Porsolt et al., 1977; Cryan and Mombereau, 2004). In this test, mice were allowed to swim for 6 min in a glass cylinder (24 × 21 cm) filled with water (23–25 °C) to a depth of 17 cm. The test was video recorded, and the time spent immobile during the last 4 min of the 6-min test was measured. Antidepressant drugs decrease immobility in this test.

### 2.6 Statistical analysis

Data are shown as mean + S.E.M. Statistical analysis was performed using the software IBM SPSS Statistics 23. Data were analyzed using two-way ANOVA for the experiment that investigated the roles of dHi, iHi and vHi on emotional behaviors under baseline conditions i.e. in the absence of fluoxetine (*Experiment 1*). The three chambers test data in Experiment 1 was analyzed using three-way ANOVA. Data from the experiment that investigated the roles of dHi, iHi and vHi on emotional behaviors under acute and chronic fluoxetine treatment were analyzed using one-way ANOVA *(Experiment 2*). When appropriate, ANOVA was followed by Fishers LSD post-hoc test for group-wise comparisons. For all comparisons, p < 0.05 was the criterion used for statistical significance.

## 3 Results

### 3.1 Lesions of the vHi (but not dHi or iHi) reduce anxiety

None of the lesions affected sociability in the three chambers test (Suppl. Fig. 1A-D), anhedonia in the saccharin preference test and female urine sniffing test (Suppl. Fig. 1E-I), or antidepressant-like behavior in the forced swim test (Suppl. Figure 1J).

**Fig. 2:**
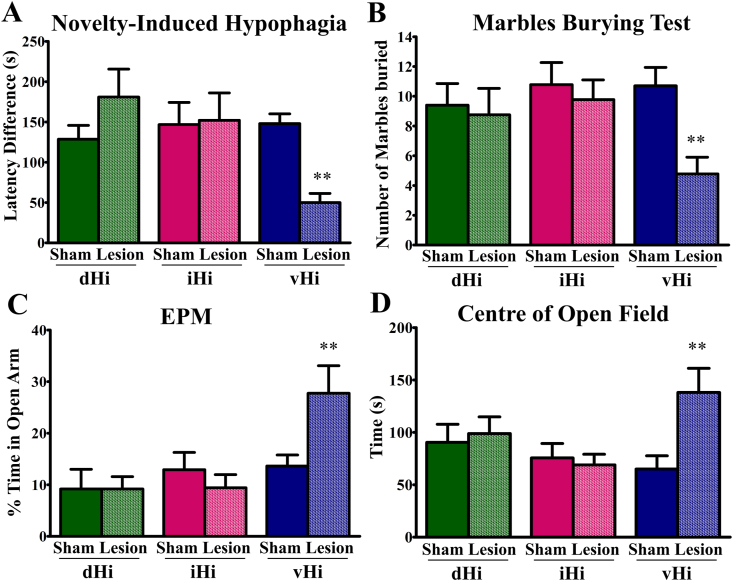
Lesions of the vHi but not the dHi or iHi reduce anxiety in several behavioral tests (A) Only vHi lesions decrease latency to drink sweetened milk in the novelty-induced hypophagia test, (B) Only vHi lesions reduce the number of marbles buried in the marble burying test, (C) Only vHi lesions increase time spent in the open arms of the elevated plus maze (EPM) and (D) Only vHi lesions increase time spent in the center of the open field **p < 0.01 compared to own sham group, according to Fishers LSD post-hoc test. N = 8–10.

Lesions of the vHi but not dHi or iHi reduced anxiety-like behavior. In the novelty-induced hypophagia test (Fig. 2A), there was a significant lesion x hippocampal sub-region interaction (F(2, 47) = 5.73, p = 0.006), and a significant effect of hippocampal sub-region (F(2, 47) = 3.65, p = 0.034). Only vHi lesions reduced latency to drink the sweet milk when compared to their corresponding sham group (p = 0.003). In the marble burying test (Fig. 2B), there was a significant lesion effect (F(1, 49) = 5.85, p = 0.019). Only vHi lesions reduced the number of marbles buried when compared to with the vHi-sham group (p = 0.005). In the elevated plus maze (Fig. 2C), there was a significant lesion x hippocampal sub-region interaction (F(2, 49) = 3.67, p = 0.033) and a hippocampal sub-region effect (F(2, 49) = 6.39, p = 0.003). Only vHi lesions increased the time spent in the open arms when compared to the vHi-sham group (p = 0.005). In the open field test (Fig. 2D), time spent in the center of the open field revealed a trend towards a lesion effect (F(1, 49) = 3.64, p = 0.062) and a significant lesion x hippocampal sub-region interaction (F(2, 49) = 3.57, p = 0.036). Only vHi lesions increased time spent in the open field center when compared to the vHi-sham group (p = 0.002). General locomotor activity was unaltered, as measured by distance travelled in the open field (Suppl. Figure 2A).

### 3.2 Only vHi lesions prevent the antidepressant-like behavioral effects of acute fluoxetine treatment

**Fig. 3:**
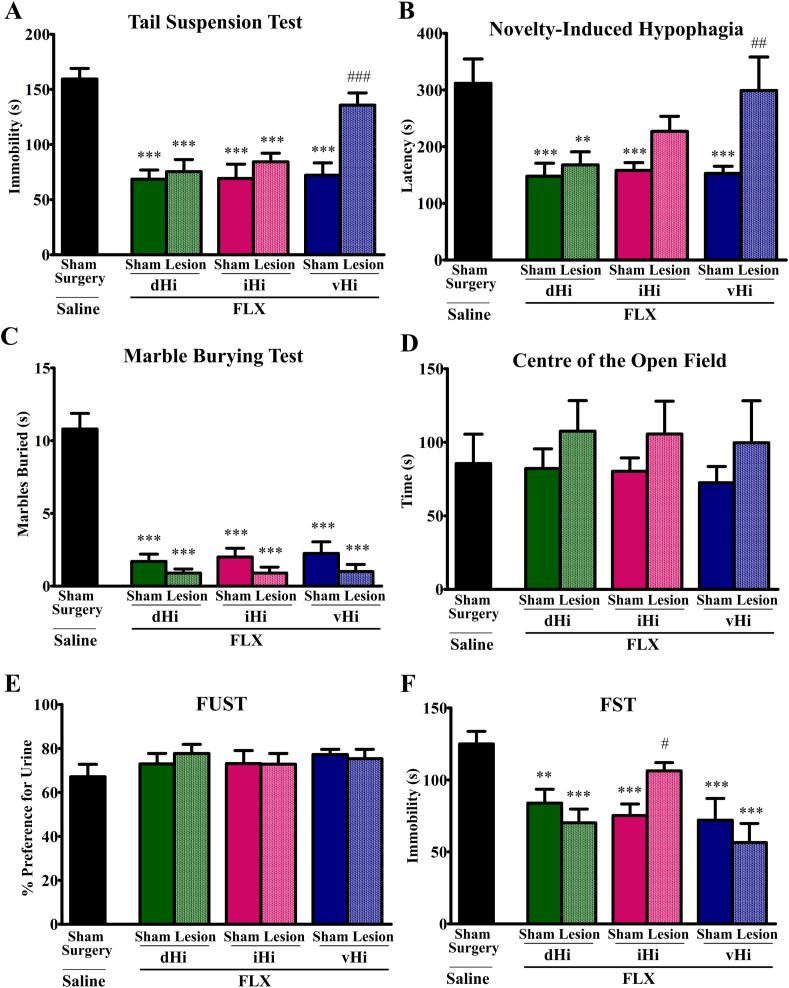
The effects of fluoxetine on anxiety-like and antidepressant-like behaviors in mice with or without dHi, iHi or vHi lesions. vHi but not dHi or iHi lesions prevented acute fluoxetine (FLX)-induced reductions in immobility time in the tail suspension test (A). vHi but not dHi or iHi lesions prevented the anxiolytic effects of chronic fluoxetine treatment in the novelty-induced hypophagia test (B). dHi, iHi or vHi lesions had no effect on chronic fluoxetine-induced decreases in the number of marbles buried in the marble burying test of anxiety (C). Chronic fluoxetine treatment did not affect time spent in the center of the open field, a measure of anxiety (D) or preference to sniff urine in the female urine sniffing test (FUST) of anhedonia (E). The reductions in immobility induced by chronic fluoxetine treatment in the forced swim test (FST) were prevented by iHi but not dHi or vHi lesions (F). **p < 0.01, ***p < 0.001 vs Sham Surgery-saline group; #p < 0.05, ##p < 0.01, ###p < 0.001 vs corresponding fluoxetine-treated sham group, according to Fishers LSD post-hoc test. N = 8–10.

In the tail suspension test (Fig. 3A), there was a significant effect of acute fluoxetine treatment (F(6, 57) = 13.02, p < 0.001). While fluoxetine decreased immobility in all groups (p < 0.001 vs sham-saline group), this effect was prevented in vHi-lesioned animals (p = 0.1 vs sham-saline group, p < 0.001 vs vHi-sham group).

### 3.3 vHi lesions prevent the anxiolytic effects of chronic fluoxetine treatment in the novelty-induced hypophagia test but not in the marble burying test nor the open field test

In the novelty-induced hypophagia test, an anxiety test sensitive to chronic antidepressant treatment (Fig. 3B), there was a significant fluoxetine effect on latency to drink milk (F(6, 58) = 4.91, p < 0.001). Chronic fluoxetine treatment reduced the latency in all sham groups when compared to the sham-saline group (dHi, p < 0.001; iHi and vHi, p = 0.001). This anxiolytic effect was unaffected by dHi lesions (p = 0.002 vs sham-saline) and attenuated by iHi lesions (p = 0.055 vs sham-saline). On the other hand, vHi lesions prevented this fluoxetine-induced anxiolytic effect (p = 0.78 vs sham-saline, p = 0.004 vs vHi-sham group).

In the marble burying test (Fig. 3C), there was a significant fluoxetine effect (F(6, 57) = 32.73, p < 0.001). Fluoxetine significantly reduced the number of marbles buried in all groups compared to the sham-saline group (all p < 0.001). Analysis of time spent exploring the center of the open field (Fig. 3D) revealed no effects of fluoxetine on this measure of anxiety (F(6, 57) = 0.53, p = 0.78). General locomotor activity was unaltered, as measured by distance travelled in the open field (Suppl. Figure 2B).

### 3.4 Only iHi lesions prevent the antidepressant effects of chronic fluoxetine treatment in the forced swim test

Chronic fluoxetine had no effects on anhedonia in the female urine sniffing test (Fig. 3E; (F(6, 58) = 1.02, p = 0.42). However, there was a significant effect of chronic fluoxetine in the forced swim test (Fig. 3F; F(6, 57) = 5.67, p < 0.001). When compared with the sham-saline group, fluoxetine decreased immobility in all other sham groups (dHi, p = 0.003; iHi, p = 0.001; vHi, p < 0.001) and this was unaffected by dHi (p < 0.001) or vHi (p < 0.001) lesions. However, iHi lesions prevented fluoxetine-induced reductions in immobility (p = 0.17 vs sham-saline, p = 0.028 vs iHi-sham group).

## 4 Discussion

We investigated, for what is to our knowledge the first time, the impact of lesioning the specific hippocampal sub-regions iHi, dHi, or vHi, on anxiety and antidepressant-like behavior in the absence or presence of the antidepressant, fluoxetine. We found that only vHi lesions reduced anxiety in the elevated plus maze, novelty-induced hypophagia, marble burying and open field tests in mice. This supports findings in rats showing that dHi lesions did not alter anxiety-like behaviors (Bannerman et al., 2002, Bannerman et al., 2003, Bannerman et al., 2004, Kjelstrup et al., 2002) but that vHi lesions reduced hyponeophagia (Bannerman et al., 2002, Bannerman et al., 2003, McHugh et al., 2004) and decreased anxiety behavior in the light-dark box (Bannerman et al., 2002, Bannerman et al., 2003, McHugh et al., 2004), in the elevated plus maze (Kjelstrup et al., 2002), in the cat-odor test (Pentkowski et al., 2006) and in the marble burying test (Wang et al., 2019). Recent studies report that stress-induced anxiogenesis was prevented by inhibition (by adult-born neurons) of a vHi population of stress-responsive cells (Anacker et al., 2018), and that the vHi together with the nucleus accumbens modulates susceptibility to depressive-like behavior (Bagot et al., 2015). Many studies however defined the vHi as the most ventral half of the hippocampus, and the dHi as the most dorsal half (Bannerman et al., 2002, Bannerman et al., 2003, Kjelstrup et al., 2002, McHugh et al., 2004) and thus included the iHi in both sub-regions. Here, we show for the first time that modulation of anxiety-like behavior is restricted specifically to the most ventral third of the hippocampus and is not impacted by iHi or dHi lesions.

The vHi is part of the tripartite anxiety network, which also includes the basolateral amygdala (BLA) and the medial prefrontal cortex (mPFC), regions that receive direct inputs from the vHi (Cenquizca and Swanson, 2007; Adhikari et al., 2011). Anxiogenic stimuli were shown to increase c-Fos expression specifically in vHi neurons that project to the BLA (Hale et al., 2008). On the other hand, optogenetic inhibition of glutamatergic projections from the BLA to the vHi in mice has been shown to reduce anxiety behavior in the elevated plus maze and open field test while stimulation of this pathway increased anxiety (Felix-Ortiz et al., 2013), but the same was not observed with the activation of the reverse pathway vHi-BLA (Jimenez et al., 2018). Additionally, optogenetic inhibition of the vHi to medial prefrontal cortex (mPFC) pathway in mice decreased anxiety in the open field test (Padilla-Coreano et al., 2016), and activation of the vHi to lateral septum pathway reduced anxiety while its inhibition increased anxiety in the elevated plus maze, novelty suppressed feeding and open field tests in mice (Parfitt et al., 2017). As illustrated above, most recent studies have focused on extra-hippocampal pathways in the regulation of anxiety rather than intra-hippocampal pathways. However, our data might also suggest that intra-hippocampal pathways also play a role and thus future studies interrogating whether intra-hippocampal pathways contribute to the behavioral effects observed here would yield further insight. Indeed, a previous study demonstrated that the inhibition of DG and CA3 neurons of the trisynaptic pathway (via knockout of α2GABAAR) in the vHi but not dHi is required for suppression of anxiety while knockout of α2GABAAR in the amygdala had no effect (Engin et al., 2016) thus suggesting that the vHi alone may be sufficient for the regulation of anxiety. Although these results reveal the essential role of the intrahippocampal circuitry in the modulation of anxiety, electrophysiological recordings of the vHi show an increased theta and synchrony with mPFC in the theta band during anxiogenic stimuli, demonstrating that vHi extra-hippocampal connectivity may be equally responsible for its effects (Adhikari et al., 2010). Taken together, we hypothesize that not only the lesion of vHi alone, but also the removal of its influences to and from other forebrain regions may underpin the anxiolytic effects observed herein.

Few studies have described the roles of hippocampal sub-regions in anhedonia or antidepressant-like behavior. Here, none of the lesions affected anhedonia (in the saccharin preference and female urine sniffing tests), or antidepressant-like behavior in the forced swim test (FST). However, it was reported that activation of the vHi to mPFC pathway mimics the antidepressant-like behavioral effects of ketamine in the rat FST (Carreno et al., 2016). These findings coupled with our observation that vHi lesions reduce anxiety in a test sensitive to chronic antidepressant treatment (novelty-induced hypophagia test), led us to hypothesize that the vHi may play a more predominant role than the dHi in antidepressant action. Thus, we next investigated the roles of each hippocampal sub-region in the antidepressant and anxiolytic effects of the antidepressant, fluoxetine.

We found that vHi lesions prevented the anxiolytic effects of fluoxetine in the novelty-induced hypophagia test but not the marble burying test. The reasons underlying these differential findings are unclear but may reflect that they measure different aspects of anxiety (e.g. the marble burying test is also a test of compulsive behavior) and involve different neural circuits (Thomas et al., 2009). Interestingly, while vHi lesions reduced anxiety in the novelty-induced hypophagia test under homeostatic conditions, vHi lesions prevented the anxiolytic effects of chronic fluoxetine treatment in this test. The reasons underlying these somewhat opposing effects are currently unclear but might relate to methodological differences between the two experiments or differences in the neural circuitry underlying regulation of anxiety behaviour in the absence or presence of antidepressant treatment. Indeed, it is possible that vHi lesions might have prevented anxiety under homeostatic conditions due to loss in the inhibitory signals to other brain regions such as the amygdala. On the other hand, the loss of the anxiolytic effects of fluoxetine in vHi-lesioned animals might be due to a loss of vHi inhibitory/excitatory connections to other forebrain regions, essential for fluoxetine's behavioral effects. It is also important to note that in the experiment investigating the impact of hippocampal lesions alone on anxiety-like behavior where animals did not receive fluoxetine treatment, animals underwent behavioral testing one week after inducing the hippocampal lesions, while fluoxetine-treated mice underwent behavioral testing four weeks after inducing hippocampal lesions. These differences in the period between lesion induction and behavioral testing might also have contributed to this counterintuitive finding. Finally, in the fluoxetine experiment, animals experienced daily intraperitoneal injections which is an additional methodological difference between the two experiments.

Both the TST and FST are highly useful tests for antidepressant drug screening (Porsolt et al., 1977; Cryan et al., 2005; Castagne et al., 2011). Here, we found that only vHi lesions prevented the effects of acute fluoxetine treatment in the tail suspension test of antidepressant-like activity (Cryan et al., 2005; O’Leary et al., 2007; O’Leary and Cryan, 2009; Liu et al., 2010). This is the first time the vHi has been shown to mediate acute behavioral effects of a first line antidepressant. On the other hand, we found that only iHi lesions prevented the antidepressant effects of chronic fluoxetine treatment in the FST which is the first time the iHi has been shown to play a role in antidepressant-like behavioral effects. These results indicate that both iHi and vHi are key brain areas for fluoxetine effects on active coping behavior. The reasons underlying the differential impact of vHi lesions in fluoxetine's effects in the TST versus the FST are unknown. However, differences may be due to differential mechanisms underlying changes in immobility induced by acute versus chronic antidepressant treatment, or may be a function of different neurobiological pathways underlying immobility behavior in the TST versus the FST (Renard et al., 2003; Airan et al., 2007). Indeed, the TST and FST can also differ in the response to the same intervention (e.g. same dose of same drug) and depending on mouse strain (Lucki et al., 2001; Cryan et al., 2005). Future studies investigating whether similar findings are obtained in the TST following chronic fluoxetine treatment and in the FST following acute fluoxetine treatment might reveal some further insight. Moreover, the roles of the hippocampal sub-regions in fluoxetine-induced anxiolytic and antidepressant effects in a chronic stress paradigm should be assessed in future experiments, as well as the possible contributions of each hippocampal sub-region in stress-induced cognitive impairment and their possible reversal by fluoxetine.

The cellular and molecular mechanisms underlying the roles of the iHi and vHi in the antidepressant- and anxiolytic behavioral effects of fluoxetine have yet to be elucidated. However, although few studies have segregated the dHi, iHi and vHi using defined neuroanatomical or genomic boundaries, recent studies propose a gene expression gradient along the hippocampal longitudinal axis (Cembrowski et al., 2016a, Cembrowski et al., 2016b, Leonardo et al., 2006, Strange et al., 2014, Thompson et al., 2008, Vogel et al., 2020). Such gradients might contribute to the diverse roles of specific hippocampal sub-regions in spatial learning & memory and anxiety. In addition, CA1 neurons from dHi, iHi and vHi have also been shown to exhibit distinct morphological and electrophysiological properties (Malik et al., 2016). Furthermore, adult hippocampal neurogenesis may also play a role. Indeed, it was reported that fluoxetine increased DCX^+^ cells in the anterior hippocampus in non-human primates (Perera et al., 2011) and that antidepressant medication increased neuroprogenitor cells in the human anterior hippocampus (Boldrini et al., 2009, Boldrini et al., 2012). Moreover, inhibition of neurogenesis in the vHi but not dHi in mice prevented the anxiolytic effects of chronic fluoxetine in a neohypophagia test (Wu and Hen, 2014). Thus, it is possible that the loss of vHi neurogenesis resultant of vHi lesions might have contributed to vHi-lesion induced impairment of fluoxetine's anxiolytic effects in the novelty induced hypophagia test. Such effects may possibly be mediated via the glucocorticoid receptor as it has been shown that antidepressants increase human hippocampal neurogenesis by activating the glucocorticoid receptor (Anacker et al., 2011), and that adult-born neurons from the vHi are more sensitive to glucocorticoid receptor stimulation than those from the dHi or iHi (Levone et al., 2020). However, given that chronic and not acute antidepressant treatment is required to increase hippocampal neurogenesis, it is unlikely that disrupted neurogenesis in the vHi can explain why vHi lesions prevented the antidepressant effects of acute fluoxetine treatment in the tail suspension test.

Chronic fluoxetine treatment decreased immobility in the FST in all sham animals, which is in agreement with previous studies in rats (Contreras et al., 2001; Vazquez-Palacios et al., 2004), and in mice (Cryan et al., 2002; Dulawa et al., 2004), including in the C57BL/6 strain (Torrisi et al., 2019). Here, we showed that the iHi was the only sub-region involved in the effects of chronic fluoxetine in the FST thus giving new insight into the functional roles of the iHi which has not been widely investigated. Further, lesions of the iHi or vHi but not dHi reduced fluoxetine-induced increases in corticosterone 30 min after FST (data not shown). Although the mechanisms underlying the contribution of the iHi to the antidepressant behavioral effects of fluoxetine in the FST remain to be elucidated in future studies, it is noteworthy that an fMRI study recently demonstrated that iHi brain connections are altered in treatment-resistant depression suggesting that neural circuits involving the iHi may be important in the clinical antidepressant response (Ge et al., 2019). Another possible mechanism may involve hippocampal neurogenesis. Interestingly, a recent study that divided the hippocampus into 12 individual sub-regions found that the effects of fluoxetine on the proliferation of hippocampal stem cells occurred specifically in the area in which the iHi transitions to the vHi, thus including both iHi and vHi subregions (Zhou et al., 2016). Thus, it might be hypothesized that fluoxetine increases neurogenesis in the iHi to produce its antidepressant effects in the FST. However, iHi lesions did not impact fluoxetine-induced behaviour in the NIH, and others have shown that fluoxetine-induced reductions in neohypophagia are dependent on adult hippocampal neurogenesis (Santarelli et al., 2003). In addition, it has been reported that the antidepressant effects of fluoxetine in the forced swim test are neurogenesis-independent in C57BL/6 mice (David et al., 2009), although in that study neurogenesis was inhibited in the whole hippocampus, and the role of neurogenesis in discrete hippocampal sub-regions was not investigated. An alternative hypothesis is that the effects of the iHi lesion could be due to a functional disconnection between the dHi and vHi and the disruption of the coherence between these other two sub-regions.

We acknowledge that the lesion approach might be considered as a potential limitation to our study Indeed, our lesions with ibotenic acid do not allow the lesion of a specific area of the hippocampus (e.g. DG or CA) nor a discrete cell type. Moreover, at present we do not know what specific vHi and iHi inputs or outputs actually mediate each of our findings. Future studies using optogenetic or chemogenetic-based approaches would assist in delineating the specific neural pathways that underlie the findings presented here. Moreover, it could be informative to ablate/inhibit adult-born neurons in the DG in each hippocampal sub-region and assess their contribution to our findings. Nevertheless, we contend that the lesion approach is still a very useful tool as a starting point to assess the functional roles of brain regions as a whole, and indeed many of the more recent chemogenetic approaches have confirmed findings from previous lesion studies, in particular those focused on the role of the vHi in the modulation of anxiety-like behaviour (Fanselow and Dong, 2010).

In summary, this study is the first to investigate the role of the iHi compared to the vHi and dHi in anxiety-like and antidepressant-like behaviors under homeostatic conditions and under antidepressant treatment conditions. We found that the vHi played a key role in anxiety and its modulation by chronic fluoxetine treatment, and both the iHi and vHi played distinct roles in fluoxetine-induced antidepressant-like behaviors. In contrast, the dHi did not play a role in antidepressant or anxiety behaviors either in the absence or presence of fluoxetine. These data suggest that both the iHi and vHi are promising targets for studies aimed at identifying novel biochemical or molecular targets for antidepressant and anxiolytic drug development, and that such future studies should focus on these specific sub-regions, rather than the hippocampus as a whole.

## Declaration of competing interest

O.F.O. and J.F.C. have received funding for unrelated contract research from Alkermes plc. O.F.O. has received renumeration from Janssen for giving an educational lecture. J.F.C. has been an invited speaker at meetings organized by Mead Johnson, Yakult, Alkermes and Janssen and has received research funding from Cremo and Nutricia. B.R.L. and G.M. reported no biomedical financial interests or potential conflicts of interest.
