# Supplementary material for: Specific sub-regions along the longitudinal axis of the hippocampus mediate antidepressant-like behavioral effects
Source: Neurobiol Stress. 2021 Apr 22;14:100331. doi: 10.1016/j.ynstr.2021.100331 (PMC8100619; doi:10.1016/j.ynstr.2021.100331)
Supplement: Multimedia component 1 [file mmc1.docx]

**Supplementary file for:**

**Specific Sub-Regions Along the Longitudinal Axis of the Hippocampus Mediate Antidepressant-like Behavioral Effects**

Running title: **Hippocampal sub-regions and antidepressant action**

Brunno Rocha Levone^1^, Gerard Moloney^1,2^, John F. Cryan^1,2#*^, Olivia F. O’Leary^1,2#*^

^1^ Department of Anatomy and Neuroscience, University College Cork, Ireland

^2^ APC Microbiome Ireland, University College Cork, Cork, Ireland.

# Equal contribution

*Corresponding authors:

Dr. Olivia F. O’Leary, MSc, PhD, Dept. of Anatomy and Neuroscience, Room 4.114, Western Gateway Building, University College Cork, Ireland. Tel: +353 (0)21 420 5480. Email: o.oleary@ucc.ie

AND

Prof. John F. Cryan, PhD, Dept. of Anatomy and Neuroscience, Room 386, Western Gateway Building, University College Cork, Ireland. Tel: +353 (0)21 420 5426. Email: j.cryan@ucc.ie

**1. Supplementary Results**

*1.1. Sociability and social novelty preference are unaffected by dHi, iHi or vHi lesions*

In the three chambers test (Suppl. Figure 1A) animals preferred to explore the chamber containing the unfamiliar animal rather than that containing the object (F(1, 94) = 179.67, p < 0.001). There was no main effect of hippocampal sub-region (F(2, 94) = 0.04, p = 0.96), lesion (F(1, 94) = 0.05, p = 0.83); nor a hippocampal sub-region x lesion interaction (F(2, 94) = 0.19, p = 0.8), nor a lesion x preference for animal interaction). Finally, there was no interaction between the three factors (F(2, 94) = 2.03, p = 0.14). (F(1, 94) = 2.87, p = 0.09). However, there was a trend towards an interaction between hippocampal sub-region x preference for animal (F(1, 94) = 2.87, p = 0.054). Post-hoc analysis revealed that each group preferred to explore the chamber containing the animal (dHi lesion group p = 0.037, all other groups p < 0.001). Interestingly, dHi-lesioned animals spent less time exploring the chamber with the animal than the dHi sham animals (p = 0.046) but still showed preference to explore the chamber containing the animal.

Animals preferred to explore the chamber containing the novel animal rather than the one containing the familiar animal (i.e. social novelty, Suppl. Figure 1B; (F(1, 94) = 45.62, p < 0.001). There was no effect of hippocampal sub-region (F(2, 94) = 0.02, p = 0.98) or lesion (F(1, 94) = 0.006, p = 0.94) nor any significant interactions [hippocampal sub-region x lesion (F(2,94) = 0.008, p = 0.99), hippocampal sub-region x preference for animal (F(2, 94) = 1.51, p = 0.23), lesion x preference for animal (F(1, 94) = 0.84, p = 0.36), hippocampal sub-region x lesion x preference for animal (F(2, 94) = 0.1, p = 0.9)]. Post-hoc analysis revealed that all groups showed a preference for the novel animal (dHi-sham, p = 0.016; dHi-lesion, p = 0.018; iHi-sham, p = 0.001; iHi-lesion, p < 0.001; vHi-sham, p = 0.009; vHi-lesion, p = 0.005), with no differences between groups in the degree of preference.

There were no effects on sociability (time spent in the chamber with the animal over the total time spent in chambers) [Suppl. Figure 1C : lesion (F(1, 48) = 2.38, p = 0.13), hippocampal sub-region (F(2, 48) = 0.96, p = 0.39) lesion x hippocampal sub-region interaction (F(2, 48) = 0.64, p = 0.53)] or on percentage preference for social novelty (time spent in the chamber with the novel animal over total time spent in chambers) [Suppl. Figure 1D; lesion (F(1, 48) = 0.61, p = 0.44), hippocampal sub-region (F(2, 48) = 0.45, p = 0.64) lesion x hippocampal sub-region interaction (F(2, 48) = 0.21, p = 0.81)].

*1.2. Anhedonia and antidepressant-like behavior are unaffected by dHi, iHi or vHi lesions*

In the saccharin preference test (Suppl. Figure 1E-H) all experimental groups preferred the sweet solution and there were no effects of any lesion at any of the time points measured [12 h, F(1, 49) = 1.52, p = 0.22; 24 h, F(1, 49) = 1.1, p = 0.3; 36 h, F(1, 49) =1.61, p = 0.21; 48 h, F(1, 49) = 1.66, p = 0.2)], hippocampal sub-region [(12 h, F(2, 49) = 2.74, p = 0.075; 24 h, F(2, 49) = 2.61, p = 0.084; 36 h, F(2, 49) = 1.05, p = 0.36; 48 h, F(2, 49) = 1, p = 0.38)], lesion x hippocampal sub-region [(12 h, F(2, 49) = 0.22, p = 0.8; 24 h, F(2, 49) = 0.11, p = 0.9; 36 h, F(2, 49) = 0.05, p = 0.96; 48 h, F(2, 49) = 0.07, p = 0.93)].

In the female urine sniffing test (Suppl. Figure 1I) all experimental groups spent more time sniffing urine than water. Two-way ANOVA revealed a significant main effect of lesion (F(1, 48) = 5.47, p = 0.024) but no main effect of hippocampal sub-region (F(2, 48) = 0.2, p = 0.82) nor a lesion x hippocampal sub-region interaction (F(2, 48) = 0.29, p = 0.75). Although there was an overall effect of lesion, subsequent post hoc analysis did not reveal any statistically significant differences between individual groups.

In the forced swim test (FST) Suppl. Figure 1J, there was no significant effects of lesion (F(1, 49) = 0.06, p = 0.81), hippocampal sub-region (F(2, 49) = 2.39, p = 0.1), nor a lesion x hippocampal sub-region interaction (F(2, 49) = 0.34, p = 0.71).

**2. Supplementary Figures**

**Suppl. Figure 1:** Lesions of the dHi, iHi or vHi do not affect sociability, preference for social novelty, anhedonia, or antidepressant-like behavior. (A) In the three-chamber test, all animals preferred to explore the chamber with the animal rather than the one with the object, although dHi lesions did reduce the time spent in the chamber with animal when compared to the dHi-sham group. (B) In the three-chamber test, all animals preferred to explore the chamber with the novel animal rather than the one with the familiar animal. (C) In the three-chamber test, the percentage preference for the animal was calculated as an index of sociability and no differences were found between groups. (D) In the three-chamber test, the percentage preference for the novel animal was calculated as an index of preference for social novelty and no differences were found between groups. (E-H) dHi, iHi and vHi lesions did not affect anhedonia as measured by the preference for saccharin in the saccharin preference test following 12 h (E), 24 h (F), 36 h (G) or 48 h (H) of saccharin availability. (I) dHi, iHi and vHi lesions did not alter anhedonia as measured by preference to sniff female urine over water in the female urine sniffing test (FUST). (J) dHi, iHi and vHi lesions did not alter immobility in the forced swim test (FST), a test of antidepressant-like behavior. *p < 0.05, ***p < 0.001, versus time spent in the chamber with the object; #p < 0.05, compared to corresponding sham group, according to Fishers LSD post-hoc test. N = 8-10.

**Suppl. Figure 2:** Neither dHi, iHi, or vHi lesions nor fluoxetine treatment alter locomotor activity in the open field test. (A) dHi, iHi and vHi lesions do not alter distance travelled in the open field. (B) Neither fluoxetine nor fluoxetine combined with hippocampal lesions alter distance travelled in the open field. N = 8-10.
